# Model-assisted ideotyping reveals trait syndromes to adapt viticulture to a drier climate

**DOI:** 10.1093/plphys/kiac361

**Published:** 2022-08-10

**Authors:** Silvina Dayer, Laurent J Lamarque, Régis Burlett, Giovanni Bortolami, Sylvain Delzon, José C Herrera, Hervé Cochard, Gregory A Gambetta

**Affiliations:** EGFV, Bordeaux-Sciences Agro, INRAE, Université de Bordeaux, ISVV, Villenave d’Ornon 33882, France; Département des Sciences de l’Environnement, Université du Québec à Trois-Rivières, Trois-Rivières, Québec, Canada G9A 5H7; Univ. Bordeaux, INRAE, BIOGECO, Cestas 33610, France; Univ. Bordeaux, INRAE, BIOGECO, Cestas 33610, France; SAVE, INRAE, BSA, ISVV, Villenave d’Ornon 33882, France; Univ. Bordeaux, INRAE, BIOGECO, Cestas 33610, France; Institute of Viticulture and Pomology, University of Natural Resources and Life Sciences (BOKU), Tulln 3430, Austria; Université Clermont-Auvergne, INRAE, PIAF, Clermont-Ferrand 63000, France; EGFV, Bordeaux-Sciences Agro, INRAE, Université de Bordeaux, ISVV, Villenave d’Ornon 33882, France

## Abstract

Climate change is challenging the resilience of grapevine (*Vitis*), one of the most important crops worldwide. Adapting viticulture to a hotter and drier future will require a multifaceted approach including the breeding of more drought-tolerant genotypes. In this study, we focused on plant hydraulics as a multi-trait system that allows the plant to maintain hydraulic integrity and gas exchange rates longer under drought. We quantified a broad range of drought-related traits within and across *Vitis* species, created in silico libraries of trait combinations, and then identified drought tolerant trait syndromes. By modeling the maintenance of hydraulic integrity of current cultivars and the drought tolerant trait syndromes, we identified elite ideotypes that increased the amount of time they could experience drought without leaf hydraulic failure. Generally, elites exhibited a trait syndrome with lower stomatal conductance, earlier stomatal closure, and a larger hydraulic safety margin. We demonstrated that, when compared with current cultivars, elite ideotypes have the potential to decrease the risk of hydraulic failure across wine regions under future climate scenarios. This study reveals the syndrome of traits that can be leveraged to protect grapevine from experiencing hydraulic failure under drought and increase drought tolerance.

## Teaser

This article quantifies the diversity of hydraulic traits across grapevine genotypes and reveals the trait combinations that can help increase grapevine drought tolerance.

## Introduction

Grapevine (*Vitis*) domestication and winemaking began more than 7,000 years ago. Over time the selection of different varieties was based on numerous criteria, for example, their growth performance, fruit characteristics, and certainly the quality of the resulting wines. Winegrape growing expanded across temperate regions. Today, climate change is making these environments hotter and dryer, increasing the frequency and/or severity of droughts (IPCC, 2014) and with that the threat of decreased yields and increased grapevine dieback. This situation is exacerbated by the fact that much of the world’s winegrape production is not irrigated (although irrigation is expanding). In this context, the selection of better drought-adapted varieties and/or genotypes is critical in reducing water use while maintaining productivity and sustainability.

Adaptation to drought involves numerous traits and trait interactions that vary within and across species (Choat et al., 2007). There is now ample evidence that plant hydraulics are the cornerstone of the physiological processes involved in plant tolerance to drought (Choat et al., 2012). However, mapping the whole plant hydraulic pathway is impossible so there is a need to identify and select key trait combinations, or “trait syndromes,” conferring better drought tolerance. Some hydraulic traits have been identified and used for predicting performance under drought (Anderegg et al., 2016; Ahrens et al., 2020). These traits include (among others) the accumulation of solutes, stomatal closure dynamics, the pressure at turgor loss, and xylem vulnerability to embolism (i.e. air bubbles resulting from cavitation; [Sperry and Pockman, 1993]). The xylem vulnerability of stems has garnered particular attention because stem embolism during severe drought ultimately leads to hydraulic failure and plant death (Brodribb and Cochard, 2009; McDowell, 2011). Thus in natural ecosystems, a genotype’s stem vulnerability is strongly linked to its ability to tolerate drought (Brodribb and Hill, 1999; Delzon and Cochard, 2014; Trueba et al., 2017).

Agricultural systems, including vineyards, seek to avoid severe droughts at all costs because of their negative effects on production. Avoiding drought in irrigated vineyards is straight forward (excluding a discussion of water resource issues). Rain fed vineyards are designed for optimum performance under drought through the choice of plant material and management strategies that maximize water availability (e.g. through lower planting densities), and minimize water use (e.g. through smaller canopies). Under current climate conditions, winegrapes rarely reach the lethal vulnerability thresholds that would induce embolism in stems, even in rain fed vineyards (Charrier et al., 2018). This is due in part to their strong vulnerability segmentation between leaves and stems, where leaves are more vulnerable to embolism and thus act as “hydraulic fuses” protecting upstream perennial organs (Charrier et al., 2016; Hochberg et al., 2016a, 2016b, 2017). Nevertheless, severe droughts that defoliate the canopy and devastate the crop are catastrophic for growers, and may produce carryover effects causing decreased vigor, lower yields, and dieback over the longer term (McDowell et al., 2008).

The identification of drought-tolerant winegrape cultivars remains obscured (Gambetta et al., 2020). One reason may be that most grapevine hydraulic traits do not vary consistently along regional moisture gradients. For example, a recent study reported mixed associations between some grapevine physiological traits and climatic regions in Europe (Bartlett and Sinclair, 2021). Some traits varied independently of climate region, and for those that did vary with climate region the quantitative relationships were not particularly strong. This is probably because in addition to climate, the selection of cultivated wine grapes has a substantial historical, regional, and cultural component. In fact, the performance of many cultivars may not always be optimum under the climatic conditions where they have been traditionally grown (van Leeuwen and Seguin, 2006).

For winegrapes, it is still unknown which trait combinations will be important in adapting viticulture to climatic change and if the trait diversity explored so far will be sufficient to tolerate a hotter and drier future. To date, only a few studies have quantified variation in functional traits within and/or across *Vitis* species (Dayer et al., 2020; Levin et al., 2020; Sorek et al., 2021), but multi-trait approaches are scarce. In a recent study, we observed that drought performance was not directly related to any single trait (e.g. maximum water use, stomatal closure, and leaf vulnerability to embolism), but instead optimum performance resulted from particular combinations between multiple traits (Dayer et al., 2020).

Adapting viticulture to a hotter and drier future will require a multifaceted approach including new management strategies, increased irrigation efficiency, and the identification and/or breeding of more drought tolerant genotypes. This study used a model-assisted multi-trait phenotyping approach to identify grapevine drought tolerant ideotypes. We characterized a wide range of hydraulic traits linked to drought tolerance in nine *Vitis* genotypes, within and across species. We then created in silico libraries of novel trait combinations by rearranging these quantified traits at random. Modeling the performance of existing cultivars along with these novel trait combinations we identified drought tolerant trait syndromes that protect grapevine from experiencing hydraulic failure under future climate change scenarios.

## Results

### The diversity of hydraulic traits within and across *Vitis* species

**Figure 1:**
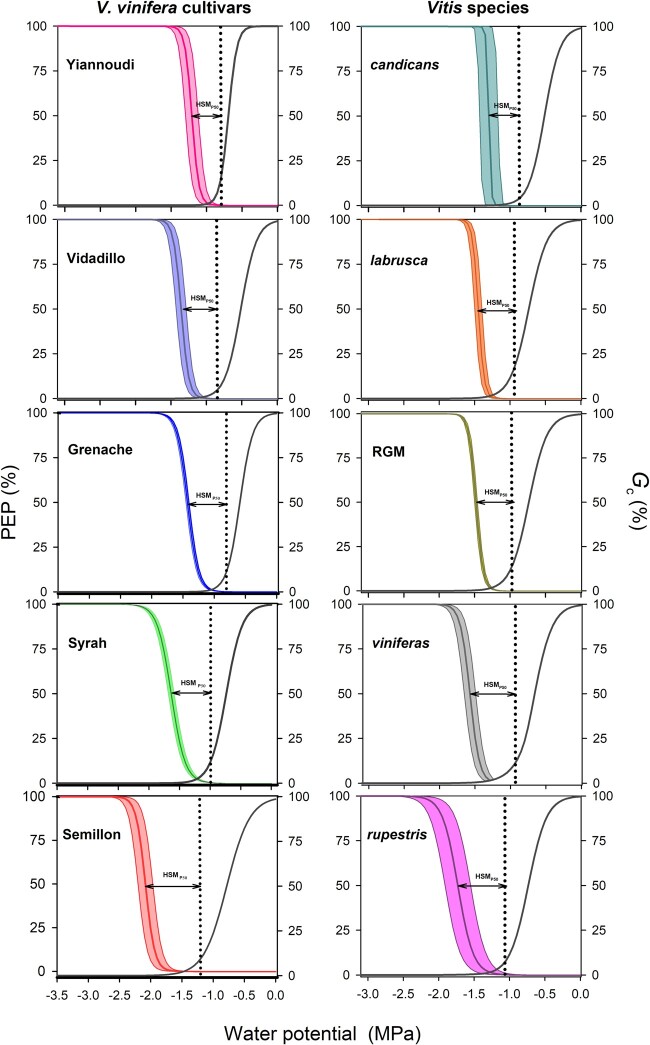
Stomatal conductance and leaf loss hydraulic conductivity depending on predawn leaf water potential. Stomatal conductance (*G*c %, gray lines) and optical hydraulic vulnerability curves (PEP %, colored lines) as a function of the leaf (*G*c %) and stem (PEP %) water potential in five *V. vinifera* cultivars (Yiannoudi, Vidadillo, Grenache, Syrah, and Semillon) and five *Vitis* species (*V. candicans*, *V. labrusca*, RGM, *V. vinifera* [mean values of all cultivars], and *V. rupestris*). The stomatal closure threshold, *G*c_90_, and the HSM_P50_ are indicated by a black dotted line and an arrow, respectively.

**Figure 2:**
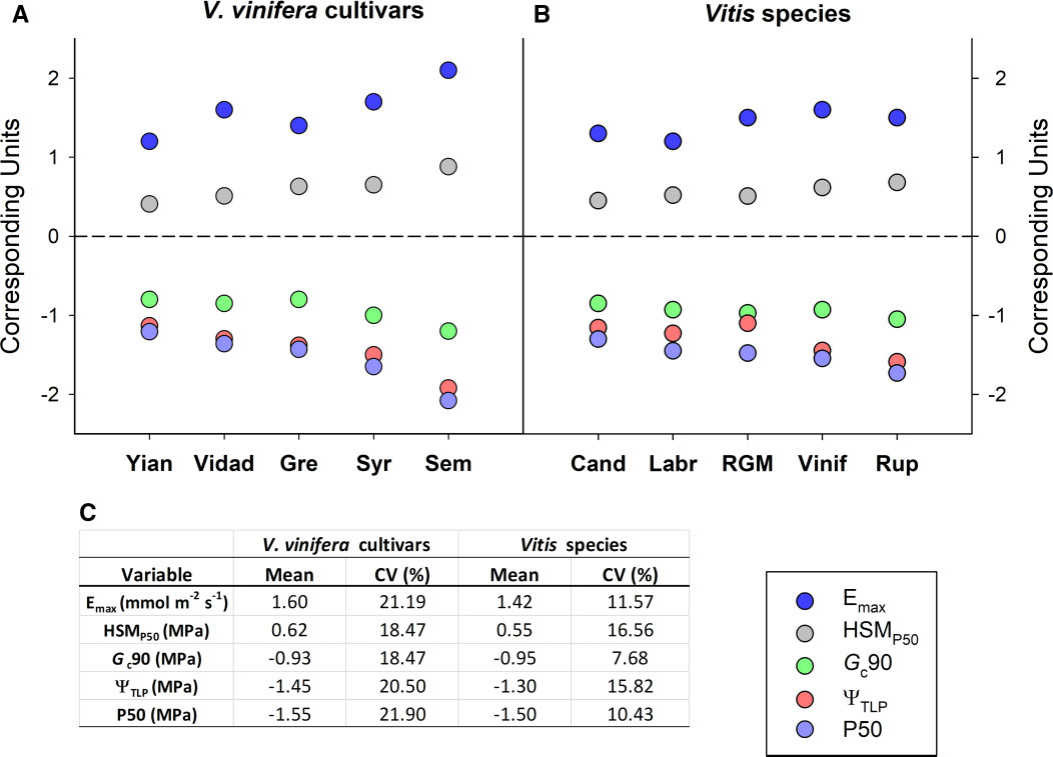
Variation of selected hydraulic traits across *V. vinifera* cultivars and species. Variation of plant maximum transpiration (*E*_max_), HSM_P50_, stomatal closure (*G*c_90_), leaf turgor loss point (Ψ_TLP_) and leaf vulnerability to embolism at P50 (P50_leaf_) across (A) five *V. vinifera* cultivars (from left to right: Yiannoudi, Vidadillo, Grenache, Syrah, and Semillon) and (B) five *Vitis* species (from left to right: *V. candicans*, *V. labrusca*, *V. riparia* (RGM), *V. vinifera* (mean values of all cultivars), and *V. rupestris*). C, Mean values and CV (CV%) for the hydraulic traits across *V. vinifera* cultivars and *Vitis* species.

We quantified a diversity of hydraulic traits that contribute to drought tolerance across nine diverse *Vitis* genotypes (five different species and five different cultivars of *Vitis vinifera*) (Figures 1 and 2 and Supplemental Data Set S1). These traits function across the full range of plant water status including traits that represent maximum water use under well-watered conditions (i.e. maximum transpiration, *E*_max_, and plant stomatal conductance, *G*c_max_), stomatal closure (*G*c_90_), the extent of osmotic adjustment (i.e. the turgor loss point, Ψ_TLP_), leaf vulnerability to embolism (P50_leaf_), and the relationship between them; the hydraulic safety margin (HSM_P__*x*_ calculated as the difference between stomatal closure and *x* percent loss of conductivity [PLC] due to embolism in the leaf, for example HSM at P12_leaf_ [HSM_P12_] or HSM at P50_leaf_ [HSM_P50_]). Comparing the dynamics of stomatal regulation and leaf vulnerability to embolism confirmed that even with variation across species, *Vitis* always maintained a positive HSM_P50_ (Figure 1 and Supplemental Data Set S2) meaning that stomata close prior to leaf embolism formation (Hochberg et al., 2016a, 2016b, 2017; Martin-StPaul et al., 2017; Creek et al., 2020). The hydraulic traits generally showed coefficients of variation (CVs) between 10% and 30% (Figure 2 andSupplemental Data Set S1). Interestingly, variation across *V. vinifera* cultivars was greater than across the species examined here for numerous hydraulic traits; *E*_max,_*G*c_90_, Ψ_TLP_ (Figure 2), leaf osmotic potential (π_0_), P12_leaf_, P88_leaf_, and HSM_P12_ (Supplemental Data Set S1). In addition, many of these traits were well-correlated with maximum water use (*E*_max_) across cultivars, but not species (Supplemental Figure S1). These observations are in agreement with previous experiments in *V. vinifera* and other angiosperms and gymnosperms where *G*c_90_, Ψ_TLP_, and P50_leaf_ were well-correlated with each other (Bartlett et al., 2016; Dayer et al., 2020), and with *E*_max_, with greater *E*_max_ corresponding to a more negative *G*c_90_, Ψ_TLP_, and P50_leaf_ (Dayer et al., 2020).

### Identifying drought tolerant trait syndromes

**Figure 3:**
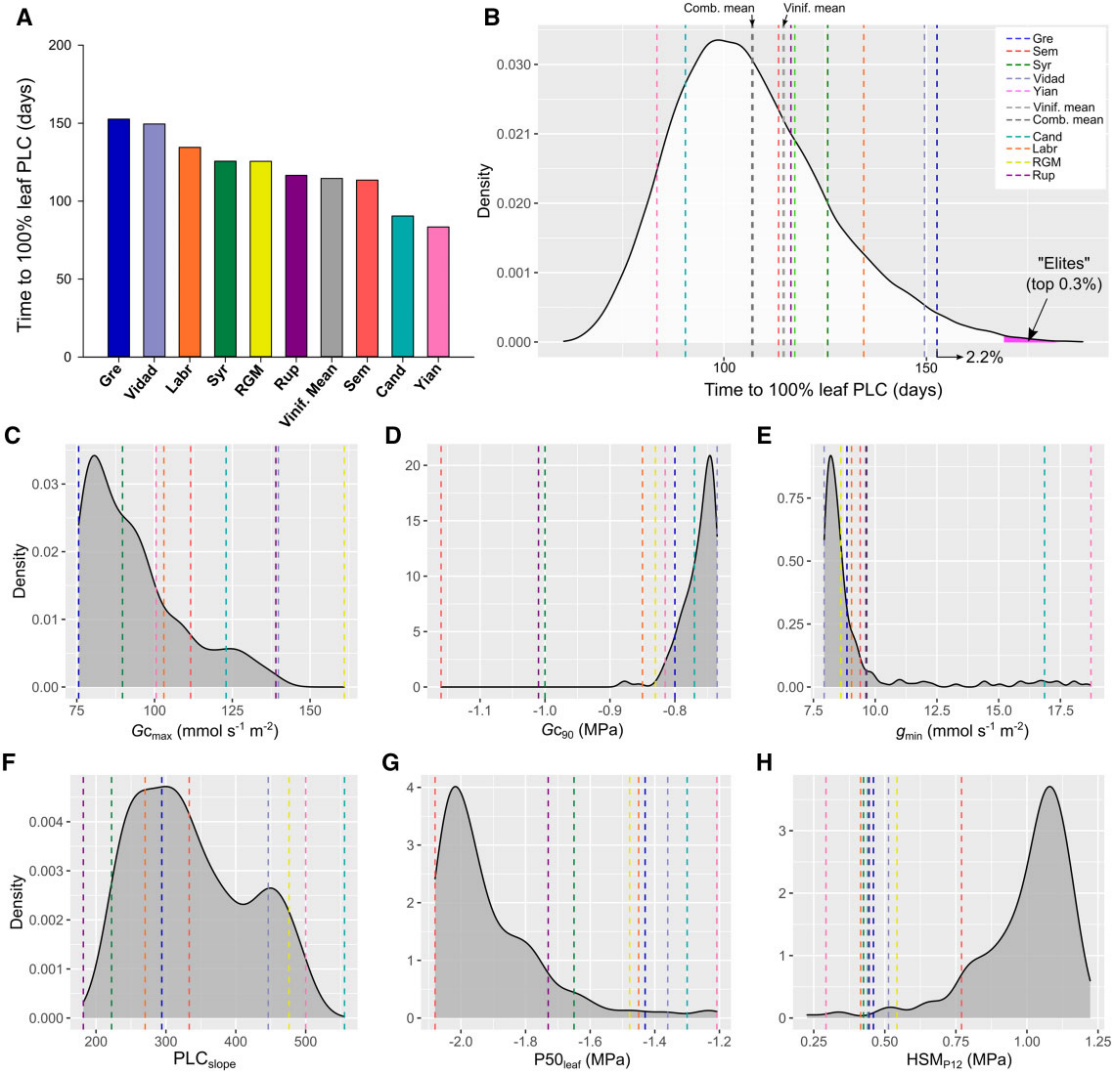
Modeled time to reach 100% loss of conductivity and traits density distributions. A, Modeled time to reach 100 PLC in leaves of the *V. vinifera* cultivars, Grenache (Gre), Vidadillo (Vidad), Syrah (Syr), Semillon (Sem) and Yiannoudi (Yian), and the *Vitis* species labrusca (Labr), RGM, Rupestris (Rup), Candicans (Cand), and *V. vinifera* mean (Vinif mean). B, Density distribution of the time to reach 100% leaf PLC for the >50,000 modeled trait combinations (created by varying eight key hydraulic traits). 2.2% of trait combinations performed better than the best performing *Vitis* genotype, Grenache. The area in pink represents the 200 best performing combinations (top 0.3%), referred to as “Elites.” C–H, Distributions (gray area) of trait values for the Elite trait combinations: (C) maximum stomatal conductance; *G*c_max_, (D) stomatal closure; *G*c_90_, (E) minimum conductance; *g*_min_, (F) the slope of leaf PLC; PLC_slope_ (G) the leaf vulnerability to embolism at P50; P50_leaf_, and (H) the HSM at P12_leaf_ HSM_P12_. The traits value for the *Vitis* genotypes is indicated by a colored dotted line.

The performance of the different genotypes under drought was evaluated using the plant hydraulic model “Sureau” (see Cochard et al., 2020; Dayer et al., 2020). We modeled the time a mature grapevine in a real vineyard scenario could last without water before reaching 100 PLC in leaves, a scenario that would result in complete canopy loss. Two *V. vinifera* genotypes, Grenache and Vidadillo outperform other varieties and species requiring 150 days to reach 100% leaf PLC, which is ca. 20–25 days more compared with the third best *Vitis labrusca* (Figure 3A and Supplemental Figure S2). We then created a library of >50,000 trait combinations by varying eight hydraulic traits (*G*c_max_, *G*c_90_, *g*_night_, *g*_min_, *π*_0_, the leaf modulus of elasticity; *ε*, P50_leaf_, and the slope of leaf PLC; PLC_slope_) at random and evaluated their performance under an identical scenario. We limited the hydraulic trait variability to the range of values we quantified for the *V. vinifera* genotypes only. This is important since crosses with non-*vinifera* species traditionally result in inferior fruit quality greatly limiting their utilization (see “Discussion”). The trait combinations varied in their time to 100% leaf PLC with a mean value of ∼107 days which was slightly lower than the *Vitis* mean of ∼115 days (Figure 3B). Only 2.2% of trait combinations performed better than the best *V. vinifera* genotype, Grenache (Figure 3B). From the modeled trait combinations, we selected the 200 best performing combinations (top 0.3%) and we refer to these as “Elites” (Figure 3B). The average time to reach 100% leaf PLC of the Elites was ∼173 days, 50% longer than the average for *Vitis*, and 13.4% longer than Grenache specifically.

We then placed the trait values of the *Vitis* genotypes within the Elite trait distributions in order to identify which traits drive superior performance. We examined only the five traits that varied more than 5% (on average) in the Elites when compared with the *Vitis* means (*G*c_max_, *G*c_90_, *g*_min_, PLC_slope_, and P50_leaf_) and the HSM_P12_ (Figure 3, C–H). The majority of Elites exhibited a drought tolerant trait syndrome with lower *G*c_max_ and *g*_min_, less negative *G*c_90_, more negative P50_leaf_, and larger HSM_P12_. There are exceptions, however, and integrating these results with the quantified *Vitis* genotypes highlights that there are a diversity of drought-tolerant trait syndromes that result in longer times to 100% leaf PLC. For example, the superior performer Grenache exhibits lower *G*c_max_ and *g*_min_, and less negative *G*c_90_, but a small HSM_P12_. In addition, having just one or several advantageous traits does not guarantee superior performance. For example, Semillon has a very negative P50_leaf_ and large HSM_P12_, but still exhibited one of the shortest times to 100% leaf PLC. Only two of the traits did not vary across the full range of measured values within the Elites; *G*c_max_ and *G*c_90_ suggesting that these traits may be critical and limiting for drought tolerance (Figure 3, C and D).

### The diversity of drought-tolerant trait syndromes

**Figure 4:**
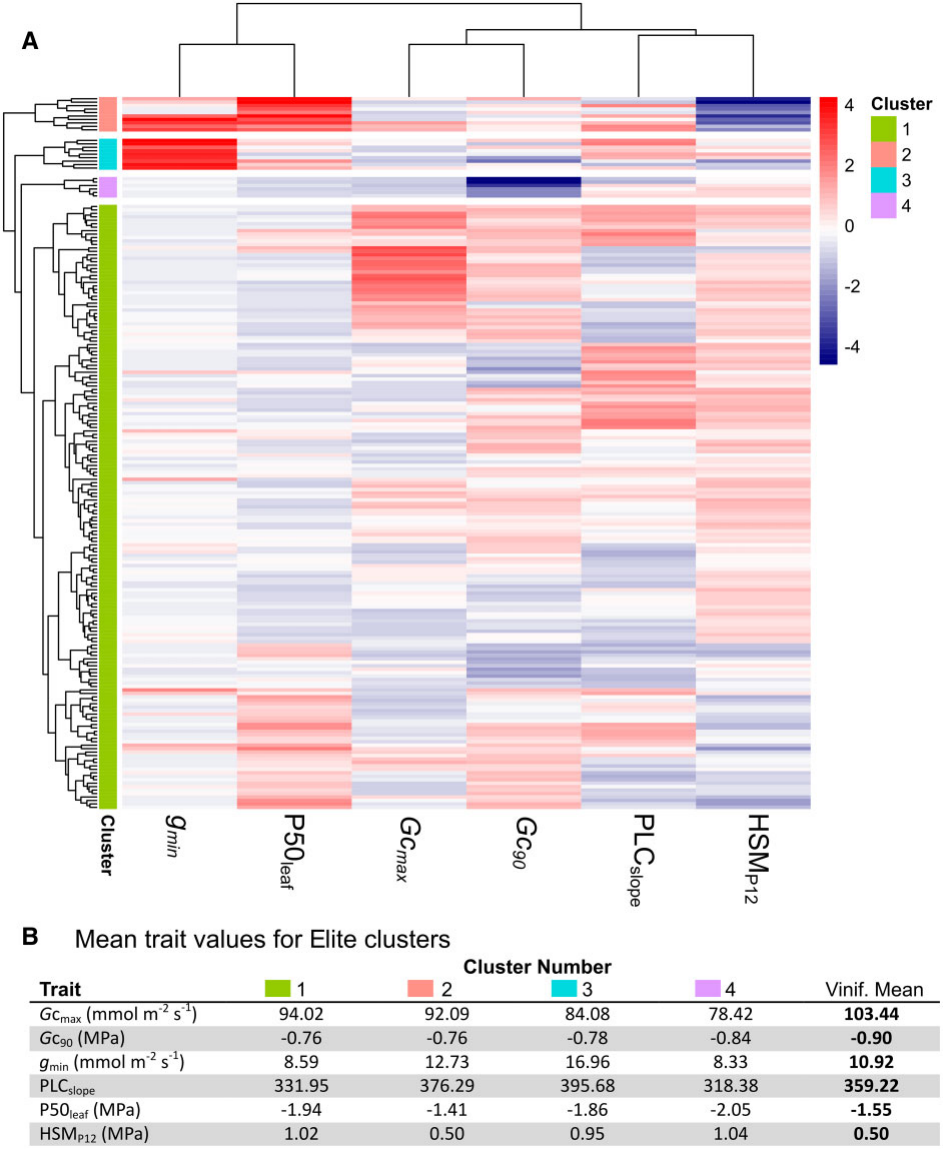
Cluster analysis and mean values of Elites. A, Cluster analysis of the Elites for six hydraulic traits: maximum stomatal conductance (*G*c_max_), stomatal closure (*G*c_90_), minimum conductance (*g*_min_), the slope of leaf PLC (PLC_slope_), the leaf vulnerability to embolism at P50 (P50_leaf_), and the HSM_P12_. Heatmap color scale represents *z*-score normalized trait values (*z*-score = observed value – mean value/standard deviation). B, Mean trait values for the four corresponding Elite clusters and average *V. vinifera* values.

Cluster analysis on the 200 best performing combinations revealed that a large diversity of trait syndromes led to the emergence of Elites (Figure 4). The optimum number of clusters was determined using the elbow method and the resulting four clusters group Elites with similar trait combinations. On the other hand, some trait combinations appeared to be very rare among Elites (Figure 4). For example, when compared with the mean *Vitis* values only the small cluster #2 exhibited similar (i.e. less negative) P50_leaf_ and correspondingly small HSM_P12_. Similarly only the small cluster #3 and members of cluster #2 achieved Elite status with high values of *g*_min_. There were exceptions to the above generalizations which reinforces the importance of the trait syndrome and not any individual trait alone.

Using Sureau modeling we created a second library of >20,000 trait combinations by randomly varying both the measured traits above (again limiting their variability to *V. vinifera* genotype values) and varying numerous other traits ±50% of their value (see Supplemental Figure S3). Expanding the varied traits resulted in a marked increase in the percentage of trait combinations with superior performance (relative to Grenache; Supplemental Figure S3). Again we selected the 200 best performing combinations and we refer to these as “Super Elites” (Supplemental Figure S3). The average time to reach 100% leaf PLC of the Super Elites was 234 days, more than double the *Vitis* mean, more than 50% longer than Grenache, and ∼35% longer than the previous Elites. Among those traits that varied more than 10% (on average) in the Super Elites (compared with the *Vitis* means) are some of the same traits identified in the Elites above such as *G*c_90_, *g*_min_, P50_leaf_, and the HSM_P12_ (Supplemental Figure S3). In addition, other important traits were identified which generally decrease the canopy surface area and increase rooting area. Notably among them are some that can be controlled to some extent by growers, such as total leaf area (and thus *E*_max_).

### Protecting against hydraulic failure under future climate change scenarios

**Figure 5:**
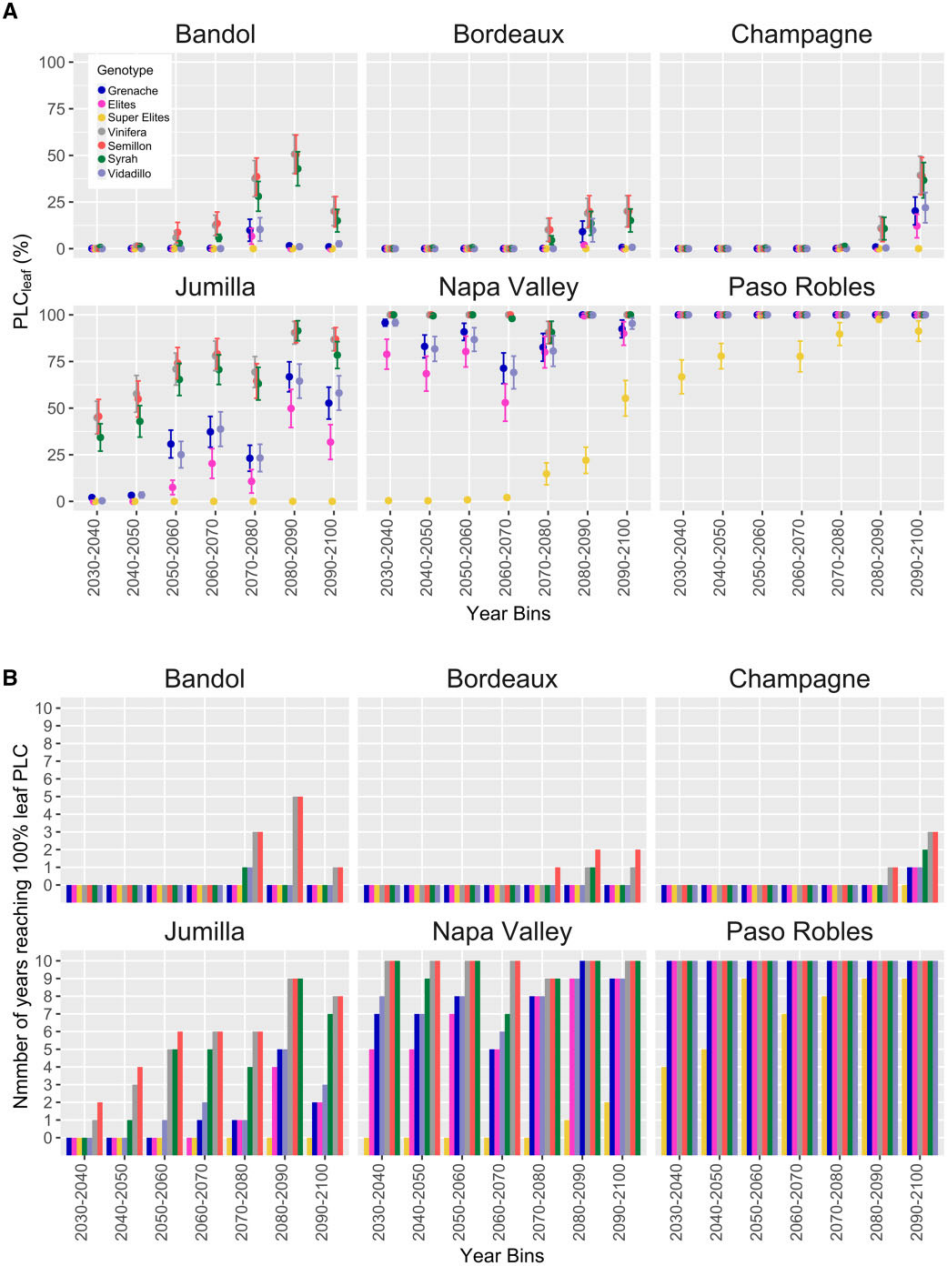
Modeled performance and trait combinations to future RCP8.5 climate scenarios 2030–2100 across different wine regions. Modeled performance of selected *Vitis* genotypes and trait combinations to future RCP8.5 climate scenarios 2030–2100 in six global wine regions: Bandol, Bordeaux, and Champagne (France), Jumilla (Spain), and Napa Valley and Paso Robles (California, USA). A, Simulated leaf PLC (%) of selected *Vitis* genotypes (Grenache, Semillon, Syrah, Vidadillo, mean *V. vinifera*), modeled Elites and Super Elites from years 2030 to 2100. Points are the means of within each 10-year bins ± standard error. All *Vitis* genotypes and year data is presented in Supplemental Figure S4. B, The number of years within each 10-year bin that *Vitis* genotypes, Elites, and Super Elites were predicted to reach 100% leaf PLC during the season.

We expanded the modeled performance of *Vitis* genotypes, Elite, and Super Elite trait combinations to future RCP8.5 climate scenarios (2030–2100) in six global wine regions: Bandol, Bordeaux, and Champagne (France), Jumilla (Spain), and Napa Valley and Paso Robles (California, USA) (Figure 5 and Supplemental Figure S4). We modeled the maximum level of leaf PLC that each genotype would experience in each year if those genotypes received no irrigation (i.e. if they were dry-farmed). In the wetter, cooler, and currently nonirrigated regions of Champagne and Bordeaux, most varieties were predicted to perform well until 2070, experiencing no, or low, levels of maximum seasonal leaf PLC (Figure 5A and Supplemental Figure S4). The high performing *Vitis* genotypes Grenache and Vidadillo were almost never predicted to reach 100 PLC (Figure 5B). Poorer performing varieties like Syrah and Semillon showed a predicted increase in maximum seasonal leaf PLC after 2070, and reached 100 PLC leaf in some years, especially after 2090 (Figure 5B). In hotter and drier regions such as Bandol and Jumilla, the predicted increase in seasonal leaf PLC occurred sooner, and in Jumilla for example even the superior performing Grenache and Vidadillo were predicted to experience canopy loss after 2050. In Jumilla, even Elite trait combinations are predicted to suffer after 2070. In warmer, hotter regions like Napa and Paso Robles in California irrigation is nearly universal currently and no current *Vitis* genotypes were predicted to survive without water (Figure 5A). Super Elite trait combinations were predicted to avoid high levels of leaf PLC well until 2080 in Napa under this dry-farmed scenario. However, in Paso Robles even the Super Elites were predicted to experience high levels of leaf PLC without irrigation.

## Discussion

Using a model-assisted multi-trait phenotyping approach, we identified drought tolerant trait syndromes that protect grapevine from experiencing hydraulic failure and increase drought tolerance under future climate change scenarios. We demonstrate that these ideotypes have the potential to greatly decrease the risk of hydraulic failure when compared with existing varieties across global wine regions under future RCP8.5 climate scenarios (2030–2100).

This study demonstrates that there is substantial variability in the hydraulic traits across *Vitis* genotypes that can be leveraged for the breeding of new genotypes. Although the number of *Vitis* species assessed here was not exhaustive, the trait diversity between *V. vinifera* cultivars was just as great as the diversity between species. Similarly, in a meta-analysis across 46 species intra-specific variation in plant hydraulic traits, such as P50, was substantial and equivalent to CV 33% of the inter-specific variability within a genus (Anderegg, 2015). This result is important because crosses within *V. vinifera* maintain fruit quality, a feature that has been a major obstacle in the adoption of other hybrid cultivars (e.g. disease resistant cultivars; [Salmon et al., 2018; Teissedre, 2018]). However, when compared across a much wider range of angio- and gymnosperm taxa the variability in hydraulic traits quantified and modeled here is quite small (Choat et al., 2012; Bartlett et al., 2016). Thus, this study demonstrates that even exploring a relatively narrow (intra-specific) range of variability can greatly enhance drought tolerance, likely resulting from emergent behaviors due to the interactions between multiple traits.

Hydraulic traits were highly correlated with each other within *V. vinifera*. This suggests that a single trait could serve as a reasonable surrogate for other traits. For example, a cultivar’s operational range of water potentials under drought could be estimated by its maximum water use under well-watered conditions (Simonin et al., 2015; Dayer et al., 2020). One critical remaining question is to what extent coordinated traits are genetically, physiologically, and/or structurally linked (Brodribb et al., 2007; Reich, 2014; Choat et al., 2018). In other words, to what extent can they be disentangled to create enhanced genotypes through breeding? In this study, the trait correlations within *V. vinifera* were absent across *Vitis* species suggesting that the traits can be disentangled to some extent.

In this study, we focused on plant hydraulics as a multi-trait system that allows the plant to maintain hydraulic integrity and gas exchange rates longer under drought. Our integration of these hydraulic traits is key in understanding a crop’s drought response and predicting performance in future climate scenarios. However, this study did not address other critically important traits for evaluating crop performance, most conspicuously fruit growth and composition. Examining the modeled total carbon assimilation we see that differences in total assimilation between varieties and the Elites are small, and that there is a substantial CO_2_ fertilization effect over time due to climate change (Supplemental Figure S5). In contrast, because Super Elites have reduced canopy surface area the model predicts reduced total assimilation as well, but only in the cooler regions. These reductions in canopy size and assimilation in the Super Elites would certainly come with decreases in productivity. These observations should not be extrapolated to fruit however. Addressing the direct effects on fruit with more sophisticated and accurate modeling will be a critical addition in predicting crop performance under drought and such attempts are already being advanced in grapevine (Zhu et al., 2018, 2019).

The model-assisted analyses in this study showed that drought tolerance cannot be accurately assessed by quantifying a single (or even several) trait(s) (Cattivelli et al., 2008; Woldesemayat et al., 2017). In general, key traits increasing tolerance tended to reduce plant transpiration and increase the HSM, but there were a multitude of drought tolerant trait syndromes across the full range of most traits. Two exceptions were maximum water use under well-watered conditions and the threshold for stomatal closure, which both appear to have hard thresholds that limit superior drought performance. Importantly, maximum water use at the parcel scale can be reduced through changing planting density (van Leeuwen et al., 2019), training systems, and/or shading (Medrano et al., 2015). This means growers can increase drought tolerance through management that does not include changing varieties or increasing irrigation.

We partly revealed the trait combinations that contribute to the traditionally observed drought tolerance of the *V*. *vinifera* cultivars Grenache and Vidadillo (indigenous to hot, dry Mediterranean regions). It is striking that only ∼2.5% of trait combinations performed better than these cultivars which is a testament to their successful historical selection by winegrape growers. Both have a very low *g*_min_ and more conservative thresholds for stomatal closure (i.e. at a less negative water potential). However, both also have relatively small HSMs, which was rare among the drought tolerant trait syndromes identified in this study.

The predictions in this study across some of the world’s top wine regions suggest that cultivars like Grenache and Vidadillo have a reduced risk of hydraulic failure when compared with other cultivars. This is critical because although the development of new cultivars is becoming more rapid, for perennial crops like grapevine it takes a substantial amount of time (van Nocker and Gardiner, 2014). Thus in the short- and medium-term, it will be important to adapt to the changing climate using existing genotypes (together with an evolution of management strategies as discussed above). However, our results also demonstrate that even with improvements in cultivar drought tolerance, irrigation is likely to become increasingly necessary depending on the region (Puy et al., 2020). Whether a region would require irrigation will be highly dependent on the total amount and distribution of rainfall throughout the season. Increased irrigation will only increase competition for fresh-water resources in regions already experiencing exceptional droughts such as California (Mann and Gleick, 2015) and the Mediterranean basin (Markonis et al., 2021).

Protecting grapevine from experiencing hydraulic failure under drought is one critical aspect of adapting viticulture to climate change and it is our hope that the trait combinations identified here can be leveraged to guide breeding programs aimed at increasing grapevine drought tolerance.

## Materials and methods

One-year-old plants of own rooted grapevines, *Vitis candicans*, *Vitis labrusca*, *Vitis Riparia Gloire de Montpellier* (RGM), *Vitis rupestris*, and five cultivars from *V. vinifera* (“Grenache,” “Semillon,” “Syrah,” “Vidadillo,” and “Yiannoudi”) from INRAE nursery (Villenave d’Ornon, France) were planted in 12 L pots containing 1 kg of gravel and 6.0 kg of commercial potting media (70% of horticultural substrate and 30% sand). Plants were grown outside under well-watered conditions for ∼2 months before the experiment started. The plants were drip irrigated with nutritive solution (NH_4_H_2_PO_4_ 0.1 mmol L^−1^; NH_4_NO_3_ 0.187 mmol L^−1^; KNO_3_ 0.255 mmol L^−1^; MgSO_4_ 0.025 mmol L^−1^; 0.002 mmol L^−1^ Fe, oligo-element [B, Zn, Mn, Cu, and Mo]) to avoid any deficiency during their development and the surface of the pots were covered with a plastic bag to prevent water loss by soil evaporation.

### Mini-lysimeter phenotyping platform experiment

Three dry-down experiments during the seasons 2018, 2019, and 2020 were conducted in the different genotypes. Experiments were carried out during the same time of the year (August–September), with vines of the same age, to control for potential developmental variability. Ten plants from each genotype were transferred to an automated mini-lysimeter greenhouse phenotyping platform where pots were continuously weighed in individual scales (CH15R11, OHAUS type CHAMP, Nänikon, Switzerland) and watered daily based on the plant weight loss by transpiration. The day before the experiment started, all the pots were irrigated until their pot capacity weight and allowed to drain overnight. A dry-down experiment was imposed by stopping irrigation on a set of five plants (*n* = 5) per species (the drying cycle lasted around 25–30 days). Five well-watered vines per genotype were kept as controls concomitantly with the water-stressed plants during the drying cycle. Air temperature, relative humidity (RH), and radiation conditions were automatically monitored every 20 min at two different positions in the greenhouse. Air temperature was maintained below ∼25°C by the cooling system of the greenhouse to avoid any heat stress. The experimental design within the platform was completely randomized.

### Balance data analysis

The transpiration per leaf area (*E* in mmol m^−2^ s^−1^) was calculated as

(1) $$E=\Delta_{w}/\mathrm{AL}/MW_{w},$$

where Δ_w_ is the rate of change in weight within the considered period (g s^−1^), AL the leaf area (m^2^), and MW_w_ the molecular weight of water (18 g mol^−1^).

The leaf area was estimated through the relationship obtained between the leaf midrib length and the leaf area, measured with a leaf area meter (LI-3000, LI-COR, Lincoln, Neb.) for each genotype using ∼100 leaves per genotype. The leaf midrib length was measured weekly on all the leaves of each plant and the respective total leaf area per plant was then calculated using the equations obtained.

The canopy stomatal conductance *G*_c_ (mmol m^−2^ s^−1^) was calculated using the simplification suggested by Monteith and Unsworth (1990):

(2) $$G_{c}\;=\;K_{G}\left(T\right).\frac{E}{D},$$

where *K_G_*(*T*) is the conductance coefficient in kPa m^3^ kg^−1^ (Ewers et al., 2001) which accounts for temperature effects on the psychrometric constant, latent heat of vaporization, specific heat of air at constant pressure, and the density of air (Phillips and Oren, 1998); *E* is the transpiration (mmol m^−2^ s^−1^); and *D* is the vapor pressure deficit (VPD) (kPa) calculated from the temperature and RH data.

The *G*_c_ values obtained were filtered by light in order to always have saturating values of radiation (photosynthetic photon flux density [PPFD] > 800 µmol m^−2^ s^−1^).

Nighttime stomatal conductance (*g*_night_) was measured with a portable open-system including an infrared gas analyzer (GFS-3000, 180 Heinz Walz GmbH, Effeltrich, Germany) equipped with CO2, humidity, temperature, and light control modules (Dayer et al., 2021).

The water status of the plants during the experiment was monitored by measuring the predawn leaf water potential (Ψ_PD_) on 3–4 plants per genotype and treatment. Measurements were performed every 2–3 days on a basal fully expanded leaf prior to any light exposure (between 5:00 A.M and 6:00 A.M.) using a “Hammel-Scholander” pressure chamber (DG Meca, Gradignan, France).

### Leaf turgor loss and minimum conductance

From the same set of plants, five plants per genotype (*n* = 5) were selected to construct the pressure–volume curves by progressively drying leaves on a laboratory bench (“bench dry method”; [Sack and Pasquet-Kok, 2011]) and measuring the Ψ_leaf_ and leaf mass at determined intervals. The plants were well-watered to pot capacity the previous day and drained overnight. In the morning (9:00–9:30 h) one healthy mature leaf (8–10th leaf from the shoot base) was cut at the base with a razor blade, sealed in a plastic bag (Whirl-Pak), and its leaf water potential (Ψ_leaf_) was measured with a “Hammel-Scholander” pressure chamber (DG Meca). The leaf mass was immediately registered in an analytical balance and the leaf was placed on a bench at room temperature (23°C) to let it dehydrate until the next Ψ_leaf_ and mass measurements. The time elapsed between measurements attempted to capture intervals of Ψ_leaf_ of 0.2–0.3 MPa until at least five points were obtained beyond the point at which zero turgor was attained.

Pressure–volume (*p*–*v*) curves were constructed by plotting the inverse of leaf water potential (−1/Ψ_leaf_) against the relative water content (RWC) which facilitated the determination of the turgor loss point as the point of transition between linear and nonlinear portions (Tyree and Hammel, 1972). Leaf RWC was calculated as: RWC = (fresh weight − dry weight)/(turgid weight − dry weight) × 100. From the *p*–*v* curves, Ψ_leaf_ at turgor loss point (Ψ_TLP_), osmotic potential at full turgor (Ψ_0_), and modulus of elasticity (*ε*) were calculated according to Bartlett et al. (2012). Leaf capacitance (*C*_leaf_) was calculated from the change in volume per change in water potential, both at full turgor (*C*_FT_), and below the turgor loss point (*C*_TLP_).

In the same set of plants used to perform the *p*–*v* curves, minimum conductance (*g*_min_; minimum water loss after stomatal closure) was determined in eight leaves by using the mass loss of detached leaves technique (Billon et al., 2019; Duursma et al., 2019). The technique consists in measuring the leaf mass loss monitored over time as the leaf dries out. Leaves from well-watered plants were detached and suspended by their petiole (to allow the lamina to transpire from both sides) in a controlled chamber (Fitoclima 1200, Aralab, Portugal) set to a constant temperature of 25°C and RH of 45%. The petioles were sealed with parafilm immediately after the cutting to avoid water losses. PPFD at the position of the samples was around 400 ± 50 µmol m^−2^ s^−1^. The mass loss of the leaves was measured every 5–10 min for the first hour and then every 15–20 min as long as the leaves dehydrated with a 0.0001 g resolution balance (Sartorius LE5201 Expert, Göttingen, Germany). The evaporation (*E*) of each sample was computed from the relationship between leaf mass and time, once water loss rates reached a steady state. The minimum conductance (*g*_min_) was calculated by using the measured VPD according to the equation *E* = *g*_min_*D*/*P* expressed in mmol m^−2^ s^−1^, where *D* is the VPD in kPa, *P* the atmospheric pressure in kPa, and *E* the transpiration normalized to the leaf area (Duursma et al., 2019).

### Noninvasive optical determination of leaf vulnerability

Leaf embolism formation and propagation were evaluated in four individuals per genotype (*n* = 4) by monitoring changes in light transmission through the xylem (Brodribb et al., 2016). Intact plants (well-watered) were placed in a room with controlled conditions at 26°C and 50% of RH and irrigation was cut-off at the beginning of the scanning. For each plant, the abaxial side of an intact mature leaf (taken from the 8th–10th node) still attached to the parent vine, was fixed on a scanner (Perfection V800 Photo, EPSON, Suna, Japan) using a transparent glass and adhesive tape. The imaged area consisted of half of each leaf including the midrib and the scanner magnification was set to give enough resolution of the midrib and at least eight major (second order) veins. Brightness and contrast as well as leaf scanned area were adjusted to optimize visualization of embolisms and provide images not exceeding 9 Mb. Each leaf was automatically scanned every 5 min throughout plant dehydration using the AutoIt 3 computer automation software.

Stem water potential (Ψ_stem_) was simultaneously measured using psychrometers (ICT International, Armidale, NSW, Australia) that were installed on the main stem of each plant located ∼10 cm above the scanned leaf to avoid inducing artifactual leaf embolism. The Ψ_stem_ values were automatically recorded every 30 min and the accuracy of the readings was confirmed by Ψ_leaf_ measurements on basal leaves previously bagged with aluminum foil (2 h at least) using a Scholander pressure bomb (DG Meca).

The stack of images captured at the end of the experiment comprised between 1,800 and 2,000 scans per leaf and was analyzed using ImageJ software and following the protocol from http://www.opensourceov.org. Briefly, total embolism was quantified by subtracting pixel differences between consecutive images (i.e. pixel values that did not change resulted in a value of zero). In these series, white pixels represented leaf embolism. Noise was removed using the ImageJ outlier removal and pixel thresholding was used to extract embolism from any background noise remaining. The embolism area per image was calculated as the sum of nonzero pixels, and the percentage of embolized pixels (PEP) at a given scan time (i.e. cumulative embolism) was determined from the total embolism area observed throughout the leaf dehydration sequence.

Vulnerability curves corresponding to the PEP as a function of stem water potential (Ψ_stem_) were fitted based on the following equation (Pammenter and Van der Willigen, 1998):

(3) $$\mathrm{PEP}=\frac{100}{1+e^{\left(\mathrm{slp}/25.\left(-P_{50_{\mathrm{leaf}}}\right)\right)}},$$

where P50_leaf_ (MPa) is the Ψ_stem_ value at which 50% of the xylem embolisms were observed and slp (% MPa^−1^) is the slope of the vulnerability curve at the inflexion point. The xylem pressure inducing the 12% (P12_leaf_) and 88% (P88_leaf_) of embolisms loss of functionality were calculated as follows: P12_leaf_ = 50/slp + P50_leaf_ and P88_leaf_ = −50/slp + P50_leaf_. One vulnerability curve was obtained per plant (*n* = 4 plants per genotype with one leaf per plant).

Finally, to visualize the dynamics of embolism spread through the leaf, spatio-temporal color maps of embolism formation were created for some of the samples by coloring the embolism area in each sequence using a color scale of Ψ_stem_ over time (Supplemental Movie S1).

### Model simulations of hydraulic traits

A soil-plant water transport model (SurEau; Cochard et al., 2020) was used to determine the predicted time it would take to reach a particular threshold (thresholds are described below) under drought. A detailed explanation of the model is given in Martin-StPaul et al. (2017) and Cochard et al. (2020). Briefly, the plant is described as a series of variable symplasmic and apoplasmic hydraulic conductances and capacitances that determine water flows and water potential along the soil–plant–atmosphere continuum. The soil hydraulic properties are defined by pedo-transfer functions following Van Genuchten (1980) that compute the soil water potential and its conductance as a function of its water content. In case of rain, the fraction not intercepted by the foliage is added to the topmost layer of the soil. When this layer reaches the field capacity point the deeper layers are rehydrated. When the three layers of soil are fully hydrated any remaining water inputs flow out of the rhizosphere and are not available to the plant. We considered the direct loss of water from the soil through evaporation to be negligible. The model computes the leaf temperature, its transpiration driven by leaf-air VPD, its regulation by stomatal closure and thus the variation in soil water content. Stomatal closure is calculated with an empirical relationship established for each variety with water potential. Beyond the point of stomatal closure, residual leaf transpiration is maintained, leading to plant dehydration and hydraulic failure under extreme water stress. Net CO_2_ assimilation was computed following Farquhar et al. (1980) and Von Caemmerer (2000), considering the effects of temperature on the model parameters. Environmental conditions were varied with day/night cycles (day: *T*_air_ = 25°C, RH_air_ = 60%, PPFD =1,500 µmol m^−2^ s^−1^, and night: *T*_air_ = 15°C, RH_air_ = 90%, PPFD = 0 µmol m^−2^ s^−1^). The modeling takes boundary layer effects into consideration by considering two conductances in series, one caused by the leaf boundary layer and one caused by the boundary layer of the canopy (Cochard et al., 2020). The effect of wind speed on these conductances was also modeled. The simulations were performed at a time step of 0.01 s and stopped when a threshold of water stress was reached corresponding to a total loss of conductivity (100 PLC) in leaves.

For the sensitivity analysis, the original SurEau model (Cochard et al., 2020) was modified so that any parameter could receive a random value in any predefined range. In a first set of simulations, we allowed the eight hydraulic parameters measured in this study (*G*c_max_, *G*c_90_, *g*_night_, *g*_min_, *π*_0_, *ε*, P50_leaf_, and PLC_slope_) to vary in the range of values observed across the *V. vinifera* cultivars. We then extended the sensitivity analysis to an additional set of parameters (Supplemental Data Set S2) allowing each of them to vary randomly ±50% of their default value. The time to leaf hydraulic failure was computed for each combination of traits under the same environmental conditions as above.

The performances of the current genotypes and improved ideotypes were tested under different climatic conditions spanning a large range of current world top wine areas. We selected six areas, three in France (Bordeaux, Champagne, and Bandol), one in southern Spain (Jumilla), and two in California (Napa Valley and Paso Robles). We selected the CNRM-CM5 RCP8.5 climatic model and extracted daily data from the INRAE SICLIMA platform for France, Cal-Adapt website for California and for Spain provided courtesy of Julien Ruffault from INRAE (Ruffault et al., 2020). For all sites, the simulations were run for a standardized the leaf area index of 1.5 m^−2^ m^−2^ and a total soil available water content of 180 mm. We simulated the 1950–2100 period assuming each year was independent of the preceding ones. Accordingly, the soil water content was re-saturated and the PLC of each organ set to zero on DOY 1. We considered that bud break occurred on DOY 120, leaves were fully expanded after 20 days, started to senesce on DOY 200, and plants were defoliated on DOY 290.

The C code of SurEau version 21-04-11 used for this study is available from the data INRAE public repository: https://doi.org/10.15454/6Z1MXK.

### Statistical analyses

Statistical analyses and fit were performed using R software (http://www.R-project.org) and GraphPad Prism version 7.00 for Windows (GraphPad Software, La Jolla, California, USA).

For the phenotyping using the mini-lysimeter platform stomatal conductance (*G*_c_), depending on predawn leaf water potential was fit according to the following sigmoid function:

(4) $$G_{c}=\frac{g_{\mathrm{sm}}}{{1+e^{\mathrm{slp}.}}^{-P_{\mathrm{gs}50}}}$$

where *g*_sm_ corresponds to maximal stomatal conductance at Ψ = 0, and slp, the sensitivity to decreasing water potential, and *P*_gs50_ the water potential inducing 50% stomatal closure.

For nonlinear regressions (*G*_c_ and *E* versus Ψ_PD_), the Akaike information criterion method was used to compare different fits.

The HSM was calculated for each genotype as the difference between the water potential at *G*c90 and the water potential.

Cluster analyses were carried out using the complete linkage method and the optimum number of clusters was determined using the elbow method.

## Supplemental data

The following materials are available in the online version of this article.

**Supplemental Figure S1.** Correlations of six hydraulic traits.

**Supplemental Figure S2.** Simulated plant stomatal conductance (*G*_c_) and loss of leaf hydraulic conductivity (PLC %) across time (days) during a soil drought (modeled using the SurEau model).

**Supplemental Figure S3.** Distribution of the time to leaf hydraulic failure in the expanded trait combinations and trait variation in the selected “Super Elites.”

**Supplemental Figure S4.** Modeled performance of *Vitis* genotypes to future RCP8.5 climate scenarios 2030–2100 in six global wine regions.

**Supplemental Figure S5.** Modeled total assimilation (*A*_tot_) to future RCP8.5 climate scenarios 2030–2100 across different wine regions: Bandol, Bordeaux, and Champagne (France), Jumilla (Spain), and Napa Valley and Paso Robles (California, USA).

**Supplemental Data Set S1.** Values of all measured hydraulic traits within and across *Vitis* species.

**Supplemental Data Set S2.** Physiological and environmental inputs for the sensitivity analysis of Vitis with the SurEau model.

**Supplemental Movie S1.** Leaf embolism formation and spread, and vulnerability curves expressed as the PEP as a function of stem water potential in three grapevine cultivars (Grenache, Semillon, and Syrah).

## Supplementary Material

kiac361_Supplementary_Data

## Notes

Si.D. and G.A.G.: conceptualization. Si.D. , L.J.L., R.B., G.B., and Sy.D.: methodology. Si.D., G.A.G., and H.C.: investigation. Si.D. and G.A.G.: visualization. G.A.G.: supervision. Si.D. and G.A.G.: writing—original draft. L.J.L., R.B., G.B., Sy.D., J.C.H., and H.C.: writing—review and editing.

The author responsible for distribution of materials integral to the findings presented in this article in accordance with the policy described in the Instructions for Authors (https://academic.oup.com/plphys/pages/general-instructions) is: Gregory A. Gambetta (gregory.gambetta@agro-bordeaux.fr).
