# Supplementary material for: Model-assisted ideotyping reveals trait syndromes to adapt viticulture to a drier climate
Source: Plant Physiol. 2022 Aug 10;190(3):1673–86. doi: 10.1093/plphys/kiac361 (PMC9614441; doi:10.1093/plphys/kiac361)
Supplement: kiac361_Supplementary_Data [file kiac361_supplementary_data.zip › PP2022RA00613DR1_PP2022RA00613DR1_Supplemental_Figures_EDITED_Clean.pdf]

Supplementary Materials

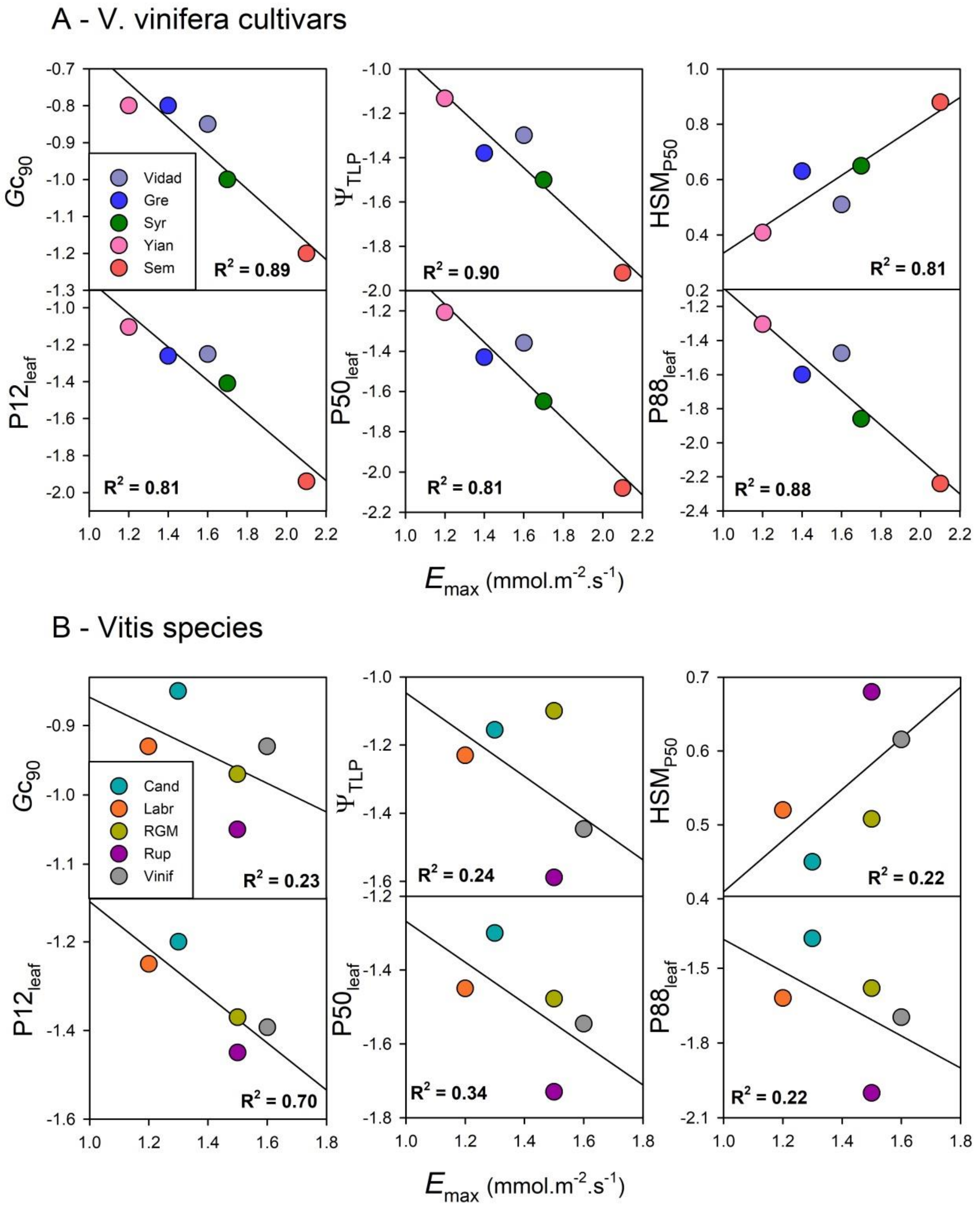

Supplemental Figure S1. Correlations of six hydraulic traits. Traits include stomatal closure; Gc_{90}, turgor loss point; Ψ_{TLP}, hydraulic safety margin at $P50_{leaf}$; (HSM_{P50}) and leaf vulnerability to embolism at $P12_{leaf}$, $P50_{leaf}$ and $P88_{leaf}$ (all units are MPa) with maximum transpiration (E_{max}) across **(A)** five *Vitis vinifera* cultivars (Gre = Grenache, Sem = Semillon, Syr = Syrah, Yian = Yiannoudi and Vidad = Vidadillo) and **(B)** five *Vitis* species (Cand = *V. candicans*, Labr = *V. labrusca*, RGM = *V. riparia*, Rup = *V. rupestris*, and Vinif = *V. vinifera*). All linear regressions across *Vitis vinifera* cultivars (panel A) are significant (P< 0.0001) , but only one linear regression ($P12_{leaf}$ versus E_{max}) is significant (P< 0.05) across *Vitis* species (panel B).

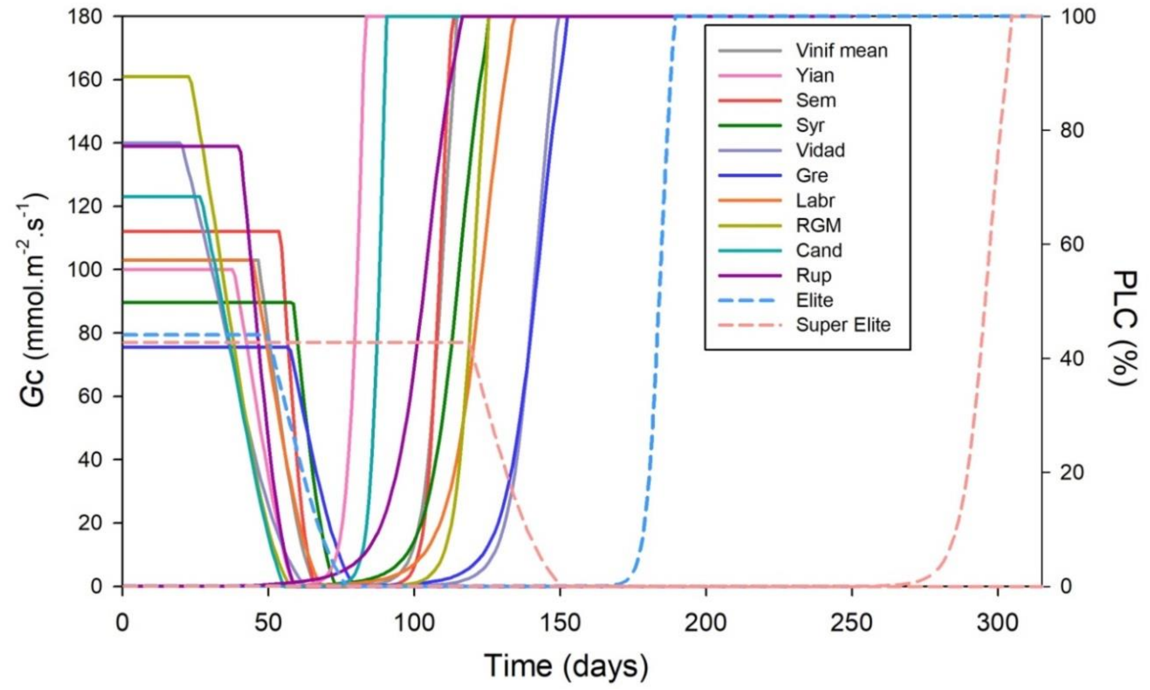

Supplemental Figure S2. Simulated plant stomatal conductance (*G*c) and loss of leaf hydraulic conductivity (PLC %) across time (days) during a soil drought (modelled using the SurEau model; (*23*)). Different colored lines represent different *Vitis* genotypes (solid colors), the top Elite trait combination (dashed light blue), and the top Super Elite trait combination (dashed pink lines). Genotype abbreviations are defined in above in Supplemental Figure S1.

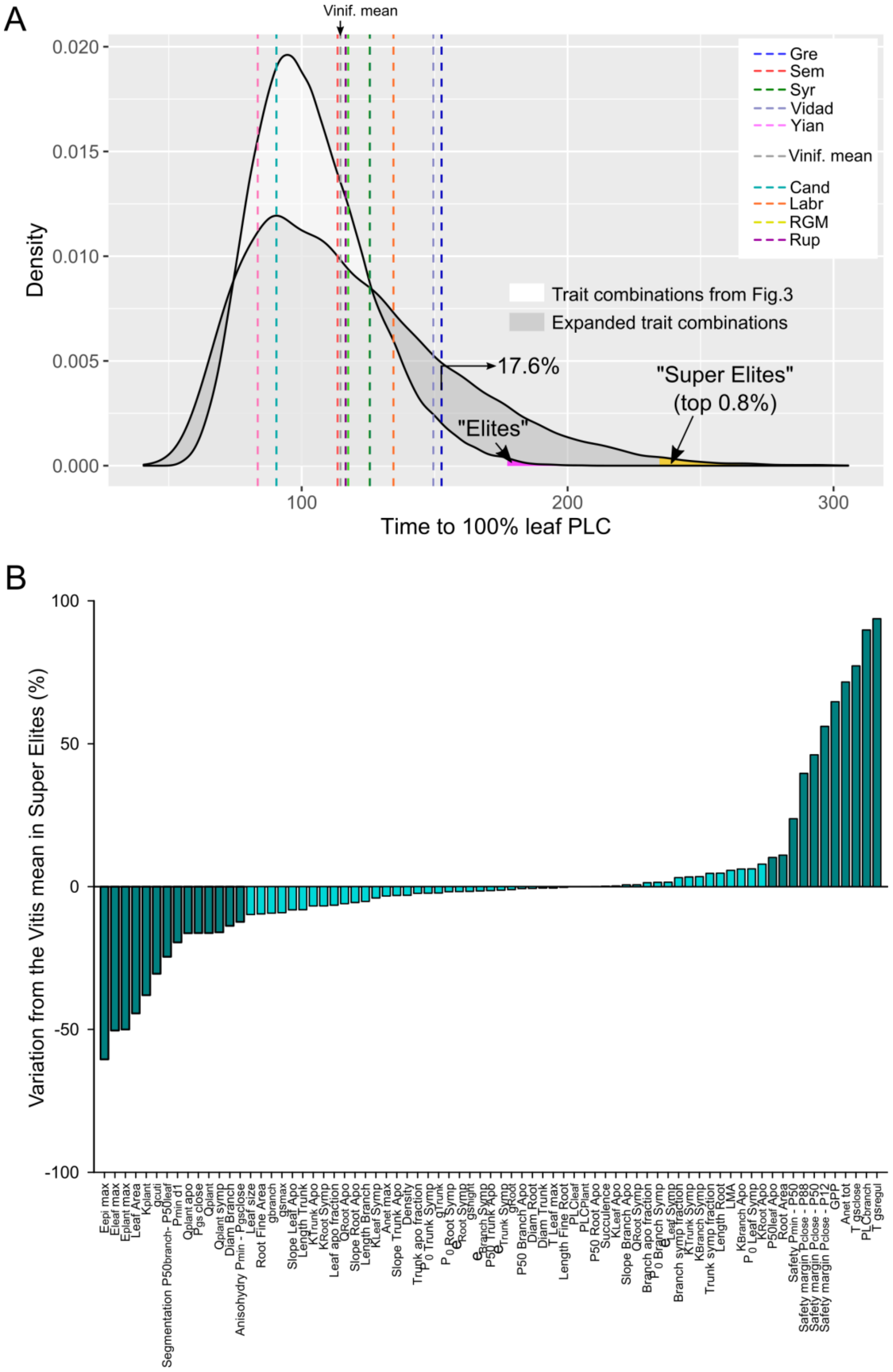

Supplemental Figure S3. Distribution of the time to leaf hydraulic failure in the expanded trait combinations and trait variation in the selected "Super Elites". **(A)** Density distribution

of the time to reach 100% leaf PLC for the modelled original trait combinations (Fig. 3B) and the expanded trait combinations (grey area). The top 200 (top 0.8%) best performing "Super Elites" are shown (yellow area). Trait values of the *Vitis* genotypes are indicated by different dotted color lines. 17.6% of expanded trait combinations performed better than the best performing *Vitis* genotype, Grenache. **(B)** Variation of the expanded traits in the "Super Elites" relative to the *Vitis* mean. Traits that varied more than 10% are colored in dark green in both extremes of the distribution. Genotype abbreviations are defined in above in Supplemental Figure S1.

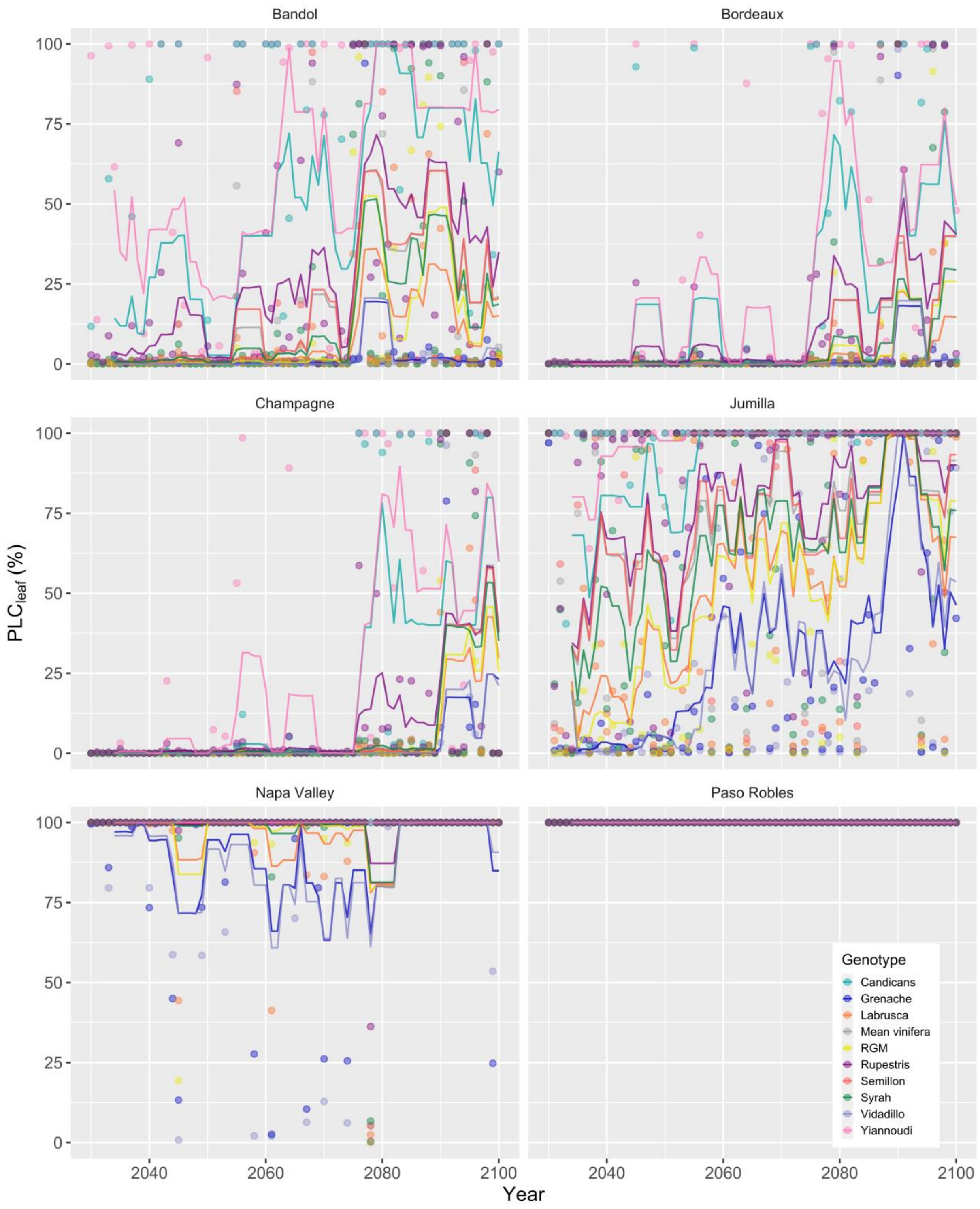

Supplemental Figure S4. Modelled performance of *Vitis* genotypes to future RCP8.5 climate scenarios 2030-2100 in six global wine regions. Dots represent the percent loss of leaf conductivity (PLC_{leaf}) in individual years and genotypes and the lines are the 5-year moving average.

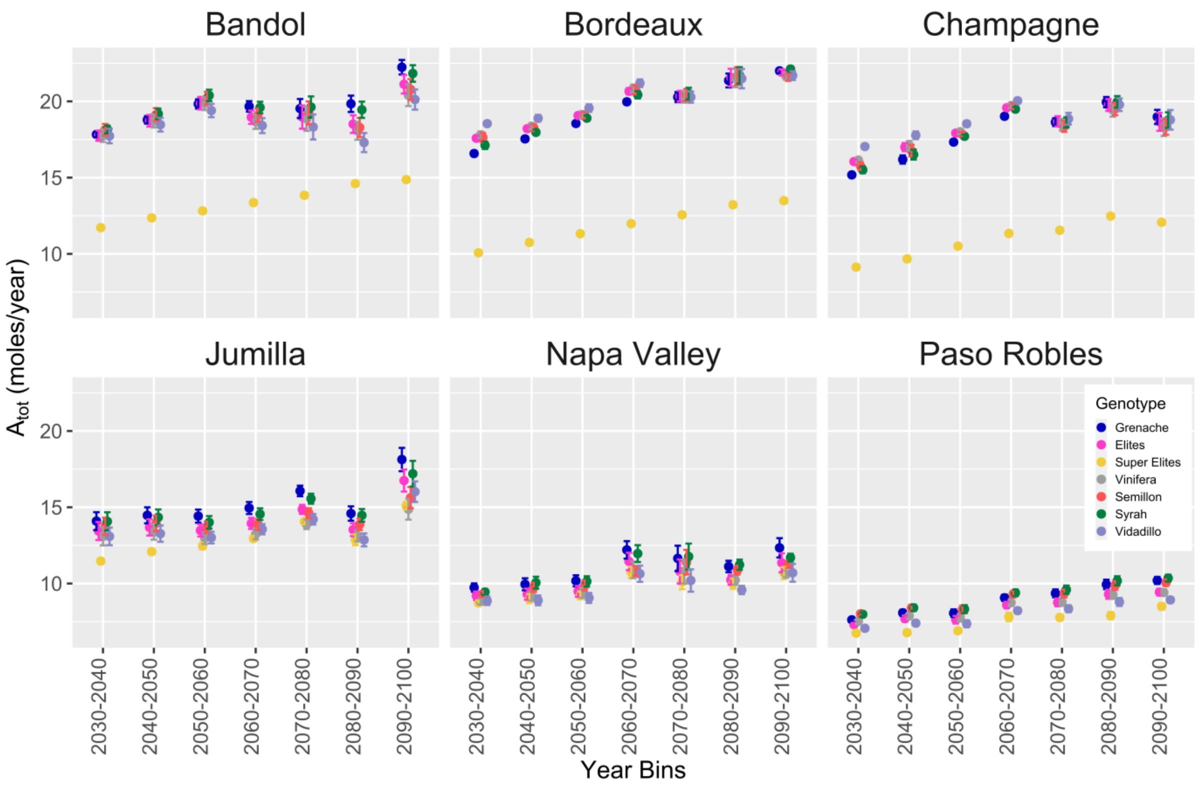

Supplemental Figure S5. Modelled total assimilation (A_{tot}) to future RCP8.5 climate scenarios 2030-2100 across different wine regions: Bandol, Bordeaux, and Champagne (France), Jumilla (Spain), and Napa Valley and Paso Robles (California, U.S.A.). Points are the means of within each 10-year bins ± standard error.
