# Supplementary material for: Model-assisted ideotyping reveals trait syndromes to adapt viticulture to a drier climate
Source: Plant Physiol. 2022 Aug 10;190(3):1673–86. doi: 10.1093/plphys/kiac361 (PMC9614441; doi:10.1093/plphys/kiac361)
Supplement: kiac361_Supplementary_Data [file kiac361_supplementary_data.zip › Supplemental Movie Legend.pdf]

Supplemental Movie Legend

Leaf embolism formation and spread, and vulnerability curves expressed as the percentage of embolized pixels (PEP) as a function of stem water potential in three grapevine cultivars (Grenache, Semillon, and Syrah).
